# Beyond mothers: the crucial role of family caregivers’ knowledge on exclusive breastfeeding in rural western China

**DOI:** 10.1186/s13006-023-00596-8

**Published:** 2023-11-06

**Authors:** Jingchun Nie, Jinbiao Ye, Shichong Wu, Nan Wang, Yangyuan Li, Yunjie Liu, Zulihumaer Reheman, Junhao Wu, Jie Yang, Yaojiang Shi

**Affiliations:** 1 Center for Experimental Economics in Education, Shaanxi Normal University, No. 620 West Chang’an Street, Chang’an District, Xi’an, Shaanxi Province China; 2 School of Economics, Xiamen University, No. 422 Siming South Road, Siming District, Xiamen, Fujian Province China

**Keywords:** Exclusive breastfeeding, Primary family caregiver, Health and nutrition knowledge

## Abstract

**Background:**

The exclusive breastfeeding rate in China remains significantly low. Numerous studies have identified the impact of maternal characteristics on exclusive breastfeeding; however, the correlation between primary family caregivers’ characteristics, such as health and nutrition knowledge, and exclusive breastfeeding still lacks clarity. The aim of this study is to investigate the association between the health and nutrition knowledge of primary family caregivers and exclusive breastfeeding in rural China.

**Methods:**

In 2019, a cross-sectional study was conducted in two prefectures within the Qinba Mountains area, located in the southern region of Shaanxi province. Data on knowledge of health and nutrition, breastfeeding practices, breastfeeding family support, breastfeeding self-efficacy, and conflict frequency were collected via structured questionnaires from 372 caregiver-infant pairs. Infant feeding practices were assessed based on the caregivers’ recall of the previous day (within the 24 h before the interview). The mother was interviewed first, followed by a brief questionnaire for the primary family caregiver, both conducted individually to minimize disruptions from other family members. Univariate and multivariate regression analyses were conducted to explore the correlation between knowledge of mothers and primary family caregivers and exclusive breastfeeding.

**Results:**

The exclusive breastfeeding rate for six-month-old infants in the sample was 15.7%. On average, mothers scored 4.6 (SD 1.4) for health and nutrition knowledge, while primary family caregivers scored 3.6 (SD 1.4). Both maternal (OR 1.48; 95% CI 1.16, 1.88) and primary family caregiver’s (OR 1.34; 95% CI 1.05, 1.70) health and nutrition knowledge were significantly associated with exclusive breastfeeding. A positive correlation (OR 1.98; 95% CI 1.40, 2.80) existed between the average health and nutrition knowledge of the mother and primary family caregiver and exclusive breastfeeding. The primary family caregiver’s health and nutrition knowledge was positively correlated with the practical family support perceived by the mother (OR 1.23; 95% CI 1.02, 1.49) and breastfeeding self-efficacy of the mother (β = 1.40; 95% CI 0.29, 2.50).

**Conclusions:**

The characteristics of the primary family caregiver play a large role in exclusive breastfeeding. To promote exclusive breastfeeding, interventions should address the needs of the whole family instead of just mothers.

**Supplementary Information:**

The online version contains supplementary material available at 10.1186/s13006-023-00596-8.

## Background

Despite the numerous advantages of breastfeeding, adherence to infant feeding recommendations remains low among mothers. Global exclusive breastfeeding rates for infants aged zero to six months were only around 44% between 2015 and 2020 [1]. However, in China, both urban and rural areas face challenges with exclusive breastfeeding rates within the first six months ranging from 20 to 30%, highlighting a significant gap towards achieving the Chinese government’s target to reach a rate of 50% by 2025 [2].

Traditionally, breastfeeding has been perceived as a responsibility primarily assigned to mothers, leading previous studies to predominantly focus on investigating the impact of maternal characteristics on breastfeeding practices. The existing literature has extensively documented several factors that exhibit a positive correlation with exclusive breastfeeding, including higher levels of education, enhanced maternal knowledge about breastfeeding, favorable attitudes towards breastfeeding, and increased self-efficacy [3–5]. Conversely, factors such as Cesarean delivery, heavy workloads or schedules, and perceived breast milk insufficiency have been identified as negative influences on the practice of exclusive breastfeeding [6–8]. Consequently, numerous interventions have been developed with a primary emphasis on supporting mothers in order to enhance exclusive breastfeeding rates.

However, there is a lack of comprehensive studies that explore how the characteristics of family members specifically influence exclusive breastfeeding, although the significance of a husband’s involvement and family support in breastfeeding has been pointed out [9–11]. It is important to note that infant care involves not only mothers but also other family members, including fathers and grandparents [12]. A study conducted in the USA suggested that grandmothers’ breastfeeding values or philosophies were found to have a positive influence on their daughters’ breastfeeding practices [13]. In southeastern China, lower education levels of grandmothers were associated with decreased rates of breastfeeding [14]. Furthermore, in rural northwestern China, it was observed that co-residence with the father was correlated with a reduced likelihood of exclusive breastfeeding due to diminished decision-making power regarding breastfeeding practices [15]. Family members involvement in parenting holds particularly true in western rural China, where the concept of a closely-knit family unit is deeply rooted, often with several generations living under the same roof and sharing parenting responsibilities. In our study, the primary family caregiver refers to individuals within the family other than the mother who assume the predominant responsibility for providing care to both the mother and infant throughout the antenatal and postnatal periods. In this particular context, grandparents are more likely to be considered as primary caregivers than fathers due to fathers’ frequent migration to urban areas for employment purposes. However, fathers are also encouraged to engage more in parenting due to their importance in children’s development [16]. Hence, it is crucial to consider the involvement of both fathers and older generations within the extended family when examining the factors that affect infant care.

The health and nutrition knowledge of family members plays a crucial role in influencing breastfeeding outcomes. On one hand, when family members possess a higher level of health and nutrition knowledge, they are more likely to provide enhanced support to mothers throughout their breastfeeding journey [17, 18]. Conversely, a knowledge gap between mothers and other family members may lead to conflicts in parenting approaches, potentially resulting in a decreased rate of exclusive breastfeeding [3, 6, 19].

Against this background, the aim of this study is to examine the relationship between health and nutrition knowledge regarding breastfeeding among primary family caregivers and the rates of exclusive breastfeeding in rural China. Furthermore, this study seeks to understand the interpersonal dynamics between caregivers within households. To achieve these goals, we employed the World Health Organization’s (WHO) definition of exclusive breastfeeding measured by recall of the previous day (within the 24 h prior to the interview) and conducted a survey among respondents from rural western China to evaluate their understanding regarding the benefits of exclusive breastfeeding and strategies for its promotion [20].

## Methods

### Setting

To investigate the health and nutritional status of mothers and infants, a cross-sectional survey was conducted in two prefecture-level cities located within the Qinba Mountains area, situated in the southern region of Shaanxi province. The Qinba Mountains area spans six provinces of western China located in Qinling and Bashan Mountains, which was one of the eight multidimensional Poverty in Regions of Contiguous Distress in 2019 [21]. In 2019, the annual per capita disposable income in the Qinba Mountains area was USD1,659.00, similar to that of rural residents in poverty-stricken counties (USD1,677.00). Our study selected all 13 of the national-level poverty-stricken counties in two prefectures in this area [22].

### Sample

The study inclusion criteria were as follows: (a) the infant resided in the sample village from birth onwards; (b) the age of the infants ranged from zero to six months (0 to 180 days). The exclusion criteria included: (a) absence or incapability of the infant’s mother to provide care for them; (b) cognitive impairment exhibited by the mother, determined by an inability to comprehend our structured questionnaire after three repetitions and verified with involvement from their families; (c) divorce or death of at least one parent. Interviews were conducted with both the mother and primary family caregiver of each infant (further details on definition provided below).

**Fig. 1:**
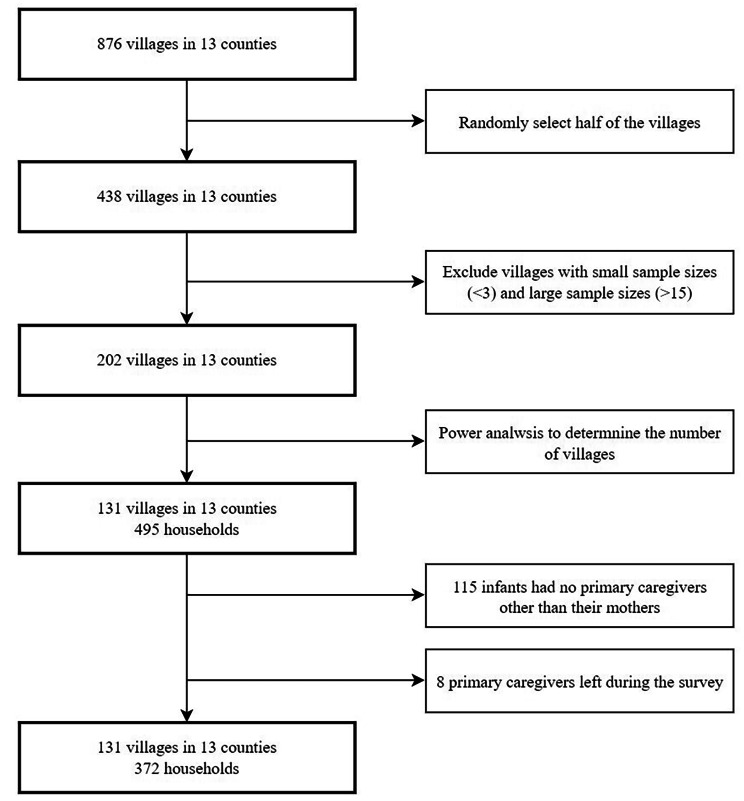
Sampling procedure and study participants

The research team employed a three-stage cluster random sampling technique to select participants for the study. Firstly, 13 nationally designated poverty-stricken counties in two prefectures within the Qinba Mountains area were chosen. Secondly, a list of villages was obtained for each county, and we randomly selected half of the total 876 rural villages from each of the 13 counties with assistance from local health commissions to form our initial village sample frame. We acquired an infant roster from the local health commissions and cross-validated it with primary healthcare providers responsible for infant management in each village. Considering the financial constraints and overall feasibility of the study, villages with a small sample size (< 3) or large sample size (> 15) were excluded, leaving 202 villages eligible for inclusion in the final sample frame. Based on our power calculation, the sample size was estimated to achieve a sampling standard error of 0.025 with a 95% confidence interval ranging from 0.10 to 0.20 for a binomial variable of 0.15, as determined from our pilot study. The final planned sample size was set at 430. Taking into account the findings from our pre-study, it was anticipated that each village would consist of approximately 3.5 households, with a 10% sample attrition rate. This led to a requirement for a minimum of 130 villages, and ultimately, 131 villages were randomly selected as the sample villages. Finally, in each of the 131 eligible villages, all caregiver-infant pairs that met the aforementioned criteria were included, resulting in a total of 495 eligible caregiver-infant pairs representing the Qinba Mountains area. However, for the purpose of analysis, 123 caregiver-infant pairs were excluded from the sample. Specifically, 115 family primary caregivers were absent during data collection and an additional 8 primary caregivers discontinued their participation after attempting to answer several questions. Consequently, a final sample size of 372 caregiver-infant pairs was considered for analysis (Fig. 1).

### Data collection

Data were collected via face-to-face interviews conducted by trained survey enumerators between March and April 2019. Enumerators received comprehensive training on the administration of survey instruments for each main component of the study, followed by a pilot study involving twenty participants to ensure research reliability and validity. Prior to the interview, eligible participants were provided with a consent form containing detailed information regarding program objectives, procedures, potential risks and benefits, as well as a privacy statement. The mother was interviewed first, following which a brief questionnaire was administered to the primary family caregiver separately in order to minimize interruptions from other family members. The survey encompassed data collection on infant and family characteristics, demographic profiles of both mothers and primary caregivers, breastfeeding practices, as well as maternal and primary caregiver health and nutrition knowledge.

### Measurements

#### Exclusive breastfeeding

Exclusive breastfeeding is the main outcome of interest. Infant feeding practices were based on the mother’s recall of the previous day (within the 24 h before the interview), including whether the infant was fed breastmilk, water, formula, fresh animal milk, juice, etc. In accordance with the definition provided by the WHO, we considered exclusive breastfeeding as feeding an infant with only breastmilk and no other liquids or solids, except oral rehydration solution and drops or syrups containing vitamins, minerals or medications [20].

#### Primary family caregivers

A questionnaire was used to collect information on all family members living in the participant’s home for longer than three months. The primary family caregiver was defined as the individual among the family members who provided the most care to both the mother and infant during the antenatal and postnatal periods, as identified by the mothers in each household. For example, if the grandmother consistently assisted with tasks such as meal preparation, diaper changes, accompanying the mother to prenatal check-ups, and offering substantial support throughout this period, she was designated as the primary family caregiver. Conversely, if the father was frequently absent due to demanding work commitments or present at home but did not actively participate in assisting with maternal and infant care, he would not be considered as fulfilling the role of primary family caregiver. Given the sample size and sample distribution, three categories of primary family caregivers were used in this study: the infant’s father, the infant’s paternal grandparents, and the infant’s maternal grandparents.

#### Health and nutrition knowledge

The mother’s knowledge of health and nutrition is the independent variable of interest, while the primary family caregiver’s knowledge in this area serves as another independent variable. Both parties employed a shared set of questionnaire items to evaluate their understanding, which was developed based on the “Guidelines for Breastfeeding in Infants Under 6 Months of Age” published by the Chinese Nutrition Society [23]. The assessment measured knowledge related to common issues within infants under six months old, including four items regarding exclusive breastfeeding, three items regarding nutrition (primarily anemia), and one item regarding general health. Questions were designed to be gender-neutral and applicable to both genders without modification. Each correct answer received one point while incorrect or unanswered questions scored zero points. Total scores ranged from 0 to 8 with higher scores indicating greater maternal nutritional and health-related knowledge. To minimize interruptions, mothers and primary caregivers responded individually.

To further investigate the association between caregivers’ health and nutrition knowledge and exclusive breastfeeding, we computed the mean level of health and nutrition knowledge as well as the disparity in such knowledge. The average level of health and nutrition knowledge among both mothers and primary family caregivers was determined by summing their respective levels of knowledge and dividing the total by two. Meanwhile, the gap in health and nutrition knowledge between mothers and primary family caregivers was calculated as the absolute difference between the mother’s level of knowledge and that of the primary caregiver.

#### Breastfeeding family support

Breastfeeding family support is one of the intermediate outcomes, which was measured using a scale designed by Zhu Xiu (2013) which contained nine items [24]. The original Cronbach’s alpha value of the scale was 0.886, indicating adequate reliability for internal consistency, and our study yielded a value of 0.891, suggesting its transferability to our study population. For each item, participants were asked to rate their level of agreement on a 5-point Likert-type scale ranging from strongly disagree to strongly agree. This scale can be divided into two dimensions: practical support which include two items (such as “my family often prepares food that is good for lactation”) and emotional support which include seven items (such as “I think my family wants me to exclusively breastfeed my child”). We calculated the mean score of each dimension and the higher score indicates stronger perceived family support. An average score of four or above represents high levels of breastfeeding family support.

#### Breastfeeding self-efficacy

Maternal breastfeeding self-efficacy also serves as an intermediate variable. The study employed the revised version of the Breastfeeding Self-Efficacy Scale-Short Form (BSES-SF), developed by Cindy-Lee Dennis in 2003 [25]. The average inter-item correlation coefficient was 0.55. The reliability of the breastfeeding self-efficacy scale was verified using Cronbach’s coefficient alpha (α = 0.94, whereas our study yielded a value of 0.62), indicating exceptional reliability and validity. This scale consists of 14 items and uses a 5-point Likert rating system, ranging from “not confident at all” to “very confident”. Higher scores indicate a higher level of maternal confidence in breastfeeding.

#### Conflict frequency

##### Conflict

frequency is another intermediate outcome. The scale used to measure conflict frequency was adapted from the work of Peterman et al. (2015) and Shroff et al. (2011) [26–28]. The scale demonstrated acceptable internal consistency reliability, with a Cronbach’s alpha value of 0.652 in our study, as recommended by Nunnally and Bernstein (1994) [28], for evaluating maternal decision-making power. We investigated disagreements within the family regarding eight household decisions related to childcare and household consumption. Participants were asked to indicate whether they had experienced differences of opinion, with “yes” scored as 1 and “no” scored as 0. Higher scores indicate a greater frequency of conflict.

#### Basic participant information

Infant and family characteristics, including the infant’s gender, age in months, number of infant siblings, and family income, were collected from the mother. Additionally, demographic information about the mother was gathered, such as maternal age, education level (senior high school and above), and health and nutrition knowledge. Primary family caregiver characteristics encompassed the age of the primary caregiver, education level (junior high school and above), relationship to the infant, and health and nutrition knowledge.

### Data analysis

STATA15.0 software was used for data cleaning and statistical analysis. Descriptive statistics were reported as frequencies (%) for categorical variables and mean (± SD) for continuous variables. Univariate and multivariate logistic regression analyses were conducted to examine the association between maternal and primary family caregiver knowledge of health and nutrition, in addition to various factors such as infant and family characteristics (infant age, number of siblings, family income), as well as maternal and primary family caregiver characteristics (age, education level), primary caregiver’s relationship to the infant, and exclusive breastfeeding status. Furthermore, potential mechanisms were explored using multivariate logistic regression models with consideration of county fixed effects. Odds ratios (ORs), corresponding 95% confidence intervals (CIs), and *P*-values were reported. Statistical significance was defined at a threshold of *p* < 0.05 for all tests.

## Results

### Sample characteristics

The descriptive statistics of the relevant variables are presented in Table 1. The sample consisted of a total of 372 caregiver-infant pairs, with boys accounting for 50.8% (189) of the infants. The mean age of the infants was 2.6 months (SD 1.8), and 40.0% were the firstborn in their households. Exclusive breastfeeding within the previous day was observed in only 15.7% (57) of the infants. The mean age of the mothers was 27.7 years, with 32.8% having attained a high school education or higher qualifications.

**Table 1:** Characteristics of participants

Variables		N (%) / mean ± SD
Exclusive breastfeeding		57 (15.7%)
**Infant and family characteristics**		
Male infant		189 (50.8%)
Infants’ age (months)		2.6 (± 1.8)
Number of siblings		0.7 (± 0.6)
Family income		3.9 (± 3.5)
**Mothers’ characteristics**		
Mothers’ age		27.7 (± 4.2)
Education (Senior high school and above)		122 (32.8%)
Health and nutrition knowledge		4.6 (± 1.4)
**Primary family caregivers’ characteristics**		
Primary family caregivers’ age		47.9 (± 11.7)
Education (Junior high school and above)		132 (35.5%)
Relationship to infant		
Father		108 (29.0%)
Paternal grandparents		219 (58.9%)
Maternal grandparents		45 (12.1%)
Health and nutrition knowledge		3.6 (± 1.4)

Amongst primary family caregivers, fathers constituted merely 29%, while grandmothers served as primary caregivers for a majority at 58.9%. The average age of primary family caregivers stood at 47.9 years, with approximately one-third possessing an educational background equivalent to junior high school or above. In terms of health and nutrition knowledge scores, mothers had an average score of 4.6 (SD 1.4), while primary family caregivers had an average score of 3.6 (SD 1.4). There was a significant difference in health and nutrition knowledge scores between mothers and primary family caregivers (t = 10.52, *p* < 0.001).

### Association between knowledge of primary family caregiver and exclusive breastfeeding

In the univariate regression analysis, both maternal (OR 1.43; 95% CI 1.16, 1.77) and primary family caregiver’s (OR 1.24; 95% CI 1.01, 1.52) health and nutrition knowledge showed a significant positive association with exclusive breastfeeding within the previous 24 h. Among other factors, low household income was significantly negatively correlated with exclusive breastfeeding (OR 0.43; 95% CI 0.19, 0.98).

The multivariate regression analysis revealed that the health and nutrition knowledge of both the mother (OR 1.48; 95% CI 1.16, 1.88) and the primary family caregiver (OR 1.34; 95% CI 1.05, 1.70) remained significantly positively associated with exclusive breastfeeding within the previous day, and the odds ratios were relatively close. Additionally, higher infant age showed a significant negative correlation with the rate of exclusive breastfeeding (OR 0.81; 95% CI 0.66, 0.98) (Table 2).

**Table 2:** Univariate and multivariate logistic regression of mothers’ knowledge and primary family caregivers’ knowledge on exclusive breastfeeding

Dependent variable: EBF	Model I: Univariate regression		Model II: Multivariate regression			
	OR (95% CI)	*P-value*	AOR (95% CI)			*P-value*
**Health and nutrition knowledge**						
Mothers’ knowledge	**1.43 (1.16, 1.77)**	**0.001**	**1.48 (1.16, 1.88)**			**0.002**
Primary family caregivers’ knowledge	**1.24 (1.01, 1.52)**	**0.038**	**1.34 (1.05, 1.70)**			**0.017**
**Infant and family characteristics**						
Infants’ age (months)	0.85 (0.72, 1.00)	0.055	**0.81 (0.66, 0.98)**			**0.032**
Number of siblings	1.04 (0.65, 1.69)	0.858	1.49 (0.78, 2.85)			0.224
Low family income	**0.43 (0.19, 0.98)**	**0.044**	0.42 (0.16, 1.06)			0.067
High family income	1.17 (0.59, 2.30)	0.655	0.89 (0.39, 2.04)			0.782
**Mothers’ characteristics**						
Mothers’ age	0.94 (0.87, 1.00)	0.066	0.95 (0.86, 1.05)			0.300
Education (senior high school and above)	1.02 (0.56, 1.86)	0.949	0.72 (0.35, 1.50)			0.385
**Primary family caregivers’ characteristics**						
Primary family caregivers’ age	1.02 (0.99, 1.04)	0.190	1.03 (0.98, 1.08)			0.219
Education (junior high school and above)	0.83 (0.45, 1.51)	0.537	0.91 (0.43, 1.94)			0.806
Relationship to infant (father as ref)						
Paternal grandparents	1.77 (0.86, 3.62)	0.120	1.21 (0.34, 4.26)			0.771
Maternal grandparents	2.26 (0.86, 5.93)	0.097	1.49 (0.35, 6.29)			0.586

Multivariate logistic regression includes county fixed effect

*AOR* adjusted odds ratio, *CI* confidence interval, *EBF* exclusive breastfeeding, *OR* odds ratio

### Potential mechanisms underlying the association

The results presented in Table 3 demonstrate a statistically significant positive correlation (OR 1.98; 95% CI 1.40, 2.80) between the average health and nutrition knowledge of both the mother and the primary family caregiver, and exclusive breastfeeding within the previous 24 h. However, no significant association was observed between the disparity in health and nutrition knowledge between the mother and primary family caregiver, and exclusive breastfeeding (OR 1.11; 95% CI 0.88, 1.39).

**Table 3:** Univariate and multivariate logistic regression of average knowledge and knowledge gap on exclusive breastfeeding

Dependent variable: EBF	Model I: Univariate regression		Model II: Multivariate regression	
	OR (95% CI)	*P-value*	AOR (95% CI)	*P-value*
**Health and nutrition knowledge**				
Average knowledge	**1.68 (1.26, 2.24)**	**< 0.001**	**1.98 (1.40, 2.80)**	**< 0.001**
Knowledge gap	1.16 (0.95, 1.43)	0.135	1.11 (0.88, 1.39)	0.383
**Infant and family characteristics**				
Infants’ age (months)	0.85 (0.72, 1.00)	0.055	**0.81 (0.66, 0.98)**	**0.033**
Number of siblings	1.04 (0.65, 1.69)	0.858	1.51 (0.79, 2.88)	0.213
Low family income	**0.43 (0.19, 0.98)**	**0.044**	0.42 (0.17, 1.07)	0.070
High family income	1.17 (0.59, 2.30)	0.655	0.91 (0.39, 2.08)	0.817
**Mothers’ characteristics**				
Mothers’ age	0.94 (0.87, 1.00)	0.066	0.95 (0.86, 1.05)	0.282
Education (senior high school and above)	1.02 (0.56, 1.86)	0.949	0.73 (0.35, 1.51)	0.393
**Primary family caregivers’ characteristics**				
Primary family caregivers’ age	1.02 (0.99, 1.04)	0.190	1.03 (0.98, 1.08)	0.220
Education (junior high school and above)	0.83 (0.45, 1.51)	0.537	0.90 (0.42, 1.91)	0.778
Relationship to infant (Father as ref)				
Paternal grandparents	1.77 (0.86, 3.62)	0.120	1.19 (0.34, 4.20)	0.787
Maternal grandparents	2.26 (0.86, 5.93)	0.097	1.47 (0.35, 6.20)	0.599
Multivariate logistic regression include county fixed effect				

*AOR* adjusted odds ratio, *CI* confidence interval, *EBF* exclusive breastfeeding, *OR* odds ratio

Table 4 shows a significant positive correlation between the health and nutrition knowledge of the primary family caregiver and the perceived practical support for breastfeeding by the mother (OR 1.23; 95% CI 1.02, 1.49). However, no significant correlation was found between the health and nutrition knowledge of the primary family caregiver and the perceived emotional support for breastfeeding by the mother (OR 0.96; 95% CI 0.80, 1.16). Furthermore, there is a significant positive correlation between the health and nutrition knowledge of the primary family caregiver and the maternal breastfeeding self-efficacy (β = 1.40; 95% CI 0.29, 2.50). However, no significant correlation was observed between the health and nutrition knowledge of the primary family caregiver and conflict frequency (β = -0.001; 95% CI -0.13, 0.12).

**Table 4:** Multiple logistic and linear regression of health and nutrition knowledge on intermediate variables

Dependent variables	Practical support		Emotional support	
	AOR (95% CI)	*P-value*	AOR (95% CI)	*P-value*
*Panel A. Multivariate logistic regression*				
Mothers’ knowledge	1.05 (0.87, 1.26)	0.641	0.98 (0.81, 1.19)	0.876
Primary family caregivers’ knowledge	**1.23 (1.02, 1.49)**	**0.027**	0.96 (0.80, 1.16)	0.704
Controls	YES		YES	
Observations	360		360	
Dependent variables	**Self-efficacy**		**Conflict frequency**	
	**β (95% CI)**	***P-value***	**β (95% CI)**	***P-value***
*Panel B. Multivariate OLS regressions*				
Mothers’ knowledge	0.81 (-0.31, 1.92)	0.156	-0.001 (-0.13, 0.13)	0.986
Primary family caregivers’ knowledge	**1.40 (0.29, 2.50)**	**0.013**	-0.001 (-0.13, 0.12)	0.987
Controls	YES		YES	
Observations	369		369	

All regressions include county fixed effect

*AOR* adjusted odds ratio, *CI* confidence interval

## Discussion

This study highlights a significantly lower prevalence of exclusive breastfeeding within impoverished rural areas in western China compared to that reported in previous literature from other regions. Exclusive breastfeeding within the first six months is only 15.7%, significantly lower than the national average of 29.2% in China [2], the global average of 43%, and the average of 37% in low- and middle-income countries [29]. Regarding family involvement in caregiving, the engagement of husbands in rural households remains notably deficient, with grandmothers chiefly shouldering the primary responsibility for caregiving. In terms of knowledge about health and nutrition, primary family caregivers demonstrate significantly lower levels of understanding compared to mothers. These findings suggest the imperative need for enhancing the capacity of family members other than mothers to deliver nurturing care.

This study highlights that exclusive breastfeeding is influenced not only by the maternal health and nutrition knowledge but also by the knowledge of key family members involved in caregiving, thereby emphasizing the multifaceted nature of its determinants. Previous research consistently demonstrates the significant impact of various maternal characteristics, including their health and nutrition knowledge, on exclusive breastfeeding outcomes [30–34]. However, this perspective has constrained the development of policies aimed at promoting exclusive breastfeeding within the first six months, as most policies have primarily targeted direct interventions for mothers. In a recent systematic review, it was found that out of the total 75 intervention arms aimed at improving breastfeeding exclusivity in low and middle-income countries, 71 primarily targeted mothers, while only four interventions involved both mothers and significant family members [35]. Neglecting the involvement of family members within the intervention may constrain its potential efficacy [36, 37]. The findings of this study suggest that enhancing the health and nutrition knowledge of primary family caregivers may potentially yield similar effects and improve breastfeeding exclusivity. Therefore, future interventions should be designed to involve additional family members, and their effectiveness should be rigorously evaluated through randomized controlled trials (RCTs) in order to promote exclusive breastfeeding.

Furthermore, the findings underscore the significance of considering the family unit as a cohesive entity rather than solely focusing on individual family members when promoting exclusive breastfeeding. The study revealed a significant correlation between the health and nutrition knowledge of both mothers and primary family caregivers and exclusive breastfeeding within the previous day. Two plausible explanations are possible. Firstly, it suggests that exclusive breastfeeding within the previous 24 h is influenced not by the knowledge of a single family member but rather by the average health and nutrition knowledge of the entire family. Secondly, it indicates the possible existence of a disparity in health and nutrition knowledge between mothers and primary family caregivers, which may potentially impact exclusive breastfeeding. The results of this study tend to support the first hypothesis over the second one, implying that both mothers and other family members play integral roles in providing nurturing care, thus emphasizing the holistic nature of exclusive breastfeeding within the context of a family unit. It further suggests that it is primarily influenced by the collective health and nutrition knowledge possessed by all members rather than solely relying on individual maternal knowledge.

While it is undeniable that the mother plays a uniquely direct role in exclusive breastfeeding, the impact of primary family caregivers should not be underestimated. However, how do primary family caregivers influence exclusive breastfeeding? Our study suggests that higher levels of health and nutrition knowledge among primary family caregivers can have a positive effect on the practical support they provide to the mother. Specifically, this knowledge enables them to assist with appropriate feeding positions, soothing techniques for the infant, attending to their needs, facilitating bathing routines, preparing lactation-promoting foods, and sharing household chores [38]. These forms of support are crucial as they enhance the mother’s belief in successfully achieving exclusive breastfeeding or her breastfeeding self-efficacy. Would improving the health and nutrition knowledge of primary family caregivers be beneficial in increasing consistency among family members’ opinions and reducing conflicts regarding exclusive breastfeeding? The results of this study do not support this assumption, thus suggesting that it may not be the main factor contributing to the low rate of exclusive breastfeeding within the previous 24 h in rural China.

The findings of this study have significant policy implications. Firstly, they underscore the urgent need to address the low rates of exclusive breastfeeding in rural western China. Secondly, they emphasize the importance of developing intervention policies that adopt a household-oriented approach to exclusive breastfeeding, going beyond the narrow focus on individual mothers. For instance, they can prioritize addressing the needs of the entire family instead of solely focusing on mothers. For home-based intervention programs, including other family members within the scope of policy interventions is significantly advantageous in terms of cost-effectiveness, as other family members are typically co-residing. It is important to note that our study holds meaningful implications across diverse cultural contexts where co-residence of multiple generations may not be the norm, as we emphasize the role of primary family caregivers beyond just grandparents.

### Limitations

Our study has a number of limitations. Firstly, due to data constraints, our study only examines the relationship between health and nutrition knowledge of primary family caregivers and exclusive breastfeeding, thereby limiting our understanding of how other characteristics of family members, such as breastfeeding attitudes, contribute to breastfeeding practices. Further comprehensive investigations are warranted to gain a deeper insight into the role of various family members in promoting exclusive breastfeeding. Secondly, it is important to note that all samples were collected from rural areas in western China, which may restrict the generalizability of our findings to other population segments.

### Electronic supplementary material

Below is the link to the electronic supplementary material.

Supplementary Material 1

## Data Availability

The datasets used and analyzed during the current study are available from the corresponding author on reasonable request.

## Abbreviations

EBF: exclusive breastfeeding
WHO: World Health Organization
