# Supplementary material for: Beyond mothers: the crucial role of family caregivers’ knowledge on exclusive breastfeeding in rural western China
Source: Int Breastfeed J. 2023 Nov 6;18:58. doi: 10.1186/s13006-023-00596-8 (PMC10626667; doi:10.1186/s13006-023-00596-8)
Supplement: Supplementary file 1 — Supplementary Material 1 [file 13006_2023_596_MOESM1_ESM.docx]

**Supplementary Information**

**Acknowledgements**

We are grateful to all respondents who participated in this study. We appreciate the enumerators for data collection efforts.

**Authors’ contributions**

J.N, J.Ya, and Y.S conceived the study. J.N, J.Ya, and N.W designed the questionnaires. J.N performed the analysis and interpreted the results with assistance from J.Y and S.W. Y.Li, Y.L, Z.R, and J.W drafted the manuscript. All authors critically revised the manuscript and approved the final manuscript as submitted.

**Funding**

This work was supported by Excellent Graduate Training Program of Shaanxi Normal University (grant LHRCCX23123), Humanities and Social Science Research Project of Ministry of Education (grant 22YJC840023), Social Science Foundation Project of Shaanxi Province (grant 2022F006), Shaanxi Province Family Education Research Project (grant JTJY2023005ZD), 111 Project (grant B16031) and Soft Science Research Project of Xi'an Science and Technology Plan (grant 23RKYJ0053).

**Availability of data and materials**

The datasets used and analyzed during the current study are available from the corresponding author on reasonable request.

**Declarations**

**Ethics approval and consent to participate**

The study was approved by the Medical Ethics Committee of Shaanxi Normal University and Xi’an Jiaotong University of China (No: 2020-1240). Each eligible participant received a consent form with information regarding programme objectives, procedures, potential risks, and benefits, as well as an explanation of privacy protection. Participants provided oral consent for inclusion in the study before engaging in a face-to-face interview with a single enumerator.

**Consent for publication**

Not applicable.

**Competing interests**

The authors declare that they have no competing interests.

**Author details**

^1^Center for Experimental Economics in Education, Shaanxi Normal University, No. 620 West Chang’an Street, Chang’an District, Xian, Shaanxi Province, China.

^2^School of Economics, Xiamen University, No. 422 Siming South Road, Siming District, Xiamen, Fujian Province, China.
